# Optimizing arginine deprivation for hard-to-treat cancers

**DOI:** 10.18632/oncotarget.22099

**Published:** 2017-10-27

**Authors:** Ramsay Khadeir, Teresa Szyszko, Peter W. Szlosarek

**Affiliations:** Peter W. Szlosarek: Center for Molecular Oncology, Barts Cancer Institute - a Cancer Research UK Center of Excellence, Queen Mary University of London, John Vane Science Center, London, UK and Barts Health NHS Trust, St Bartholomew’s Hospital, London, UK

**Keywords:** arginine, cancer, ASS1, arginine depletion, resistance

Arginine-dependent cancers represent a significant fraction of malignancies characterized by loss of urea-cycle enzymes, especially argininosuccinate synthetase (ASS1) and argininosuccinate lyase (ASL). As a tumor suppressor ASS1 catalyzes the condensation of aspartate and citrulline into argininosuccinate, and impacts multiple biological pathways involving arginine either directly, for instance via nitric oxide or mTOR, or indirectly, such as modulation of nucleotide synthesis [1, 2]. Loss of ASS1 promotes increased tumor cell proliferation and invasion, and is immunosuppressive, all attributes of a highly tumorigenic cancer [3, 4]. ASS1 deficiency has been identified across the spectrum of haematological, epithelial and mesenchymal tumors, however regulation and expression of the enzyme displays significant variability and is tissue-specific. This is most evident under arginine withdrawal, which has been explored for several decades as a novel metabolic anticancer therapy targeting the arginine-dependent phenotype. Methylation-dependent silencing of the ASS1 promoter reported in mesothelioma and bladder cancer cell lines confers exquisite sensitivity to the arginine-lowering agents, arginine deiminase or arginase. In contrast, ASS1 is induced rapidly in tumor cell lines without ASS1 promoter methylation limiting the applicability of arginine deprivation under these circumstances, particularly as a monotherapy. ASL, which is downstream of ASS1 and converts argininosuccinate into arginine and fumarate, has a secondary role in modulating tumoral arginine auxotrophy and sensitivity to arginine depletors in cancers including glioblastoma multiforme [5].

Arginine deprivation entered the clinic over a decade ago with pegylated arginine deiminase (ADI-PEG 20), which catalyzes the conversion of arginine into citrulline and ammonia, thereby recycling the former into arginine in ASS1 competent cells. Several monotherapy cancer studies of ADI-PEG 20 revealed safety and promising early activity despite the antigenic properties of a mycoplasma-derived enzyme. However, a recent phase 3 study of ADI-PEG 20 versus placebo in patients with post-sorafenib relapse in liver cancer was negative for overall survival. Post-hoc analyses revealed that ASS1 was upregulated by sorafenib and may have influenced patient outcome (clinicaltrial.gov identifier NCT01287585). In contrast, a modest improvement in progression-free survival in a randomized phase 2 study in patients with ASS1-deficient mesothelioma versus best supportive care alone was reported in the ADAM trial highlighting a need for patient selection in future studies [6]. Early phase clinical studies of several non-antigenic pegylated-arginases are underway and further testing will reveal how the differential catalysis of arginine into ornithine and urea will impact tumorigenesis.

In addition to ASS1 selection several groups have used molecular imaging to investigate responses to ADI-PEG 20. The increased glycolytic activity of neoplastic cells (Warburg effect) led to the development of fluoro-2-deoxy-D-glucose positron emission tomography (FDG-PET/CT). Early metabolic responses with FDG-PET/CT to arginine depletion have been evaluated in the ADAM trial for mesothelioma using EORTC based recommendations for change in standardized uptake value (SUVmax) to define the response categories (progression (PD)> 25% increase and partial metabolic response (PMR) >15% decrease in SUVmax). Whilst no modified RECIST partial or complete responses were observed during the study, FDG-PET/CT revealed a PMR in 46%; stable disease in 31%; a mixed response in 8% and progressive disease (PD) in 15% of patients [6]. However, ADI-PEG 20-induced arginine deprivation is known to increase serine biosynthesis, glutamine anaplerosis, oxidative phosphorylation, and decrease aerobic glycolysis, effectively inhibiting the Warburg effect, thereby limiting the wider applicability of FDG-PET/CT [7]. The reduction of glycolysis in cells otherwise dependent on aerobic glycolysis is correlated with reduced PKM2 expression and phosphorylation and upregulation of phosphoglycerate dehydrogenase (PHGDH). Recent work has investigated fluoro-3’-deoxythymidine (FLT)-PET/CT, a proliferation surrogate, as a potentially more robust response biomarker to arginine deprivation therapy, which overcomes the pitfalls of FDG-PET/CT. In preclinical studies, it has been shown that the combination of arginine deprivation and pemetrexed leads to a potentiation of cytotoxicity in ASS1-negative tumor cells and is accompanied by suppression of de novo thymidine synthesis with decreased levels of thymidylate synthase and the salvage pathway via reduced thymidine kinase 1 [3]. Recent clinical work shows an early and end of treatment response on FLT PET/CT, which is at least as good as RECIST response. Indeed, early phase combination trials of arginine deprivation with chemotherapy are reporting increased efficacy, and sustained arginine depletion with reduced immunogenicity of ADI-PEG 20 [8]. In thoracic cancers this multimodality strategy has instigated the phase 2/3 ATOMIC-meso trial of pemetrexed and cisplatin with or without ADI-PEG 20 focusing on chemorefractory (non-epithelioid) mesotheliomas.

**Figure 1:**
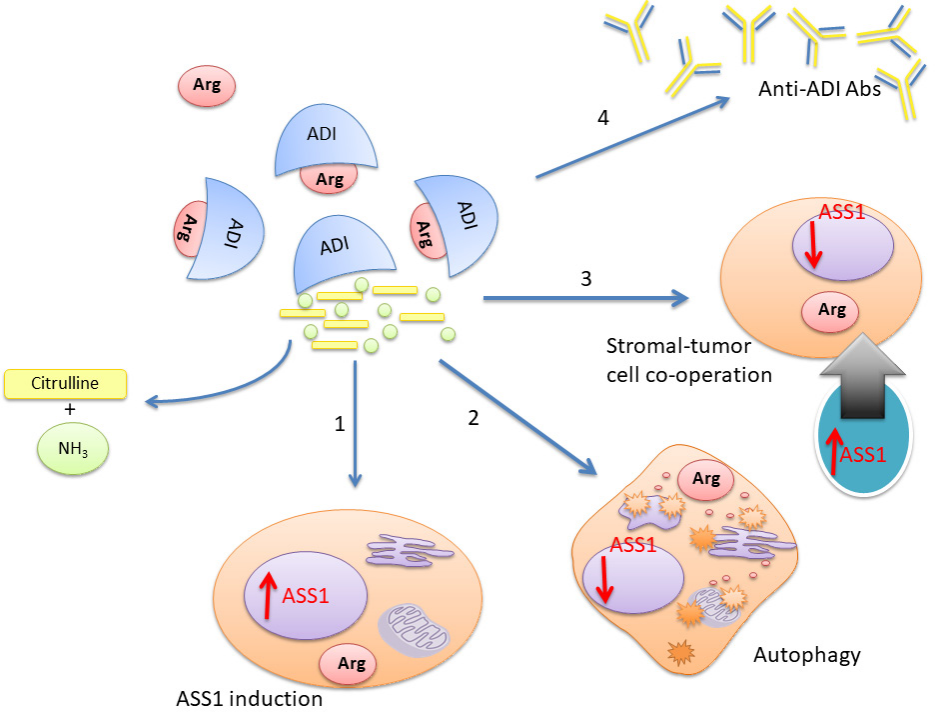
Emerging resistance to arginine deprivation in cancer Several mechanisms may reduce the efficacy of arginine depleting enzymes for cancer therapy: (1) ASS1 upregulation; (2) autophagy; (3) stromal-tumor cell metabolic co-operation; (4) and anti-drug antibodies (e.g. to ADI-PEG 20). Some approaches to overcoming resistance include combining arginine depletors with chemotherapy, human (non-antigenic) arginases, and autophagy modulators such as chloroquine.

Lastly, further optimization of arginine deprivation for cancer therapy may be achieved with modulation of key resistance pathways (Figure 1). As noted upregulation of ASS1 is a significant limitation with a potentially narrow therapeutic index for pharmacological intervention, and is also variable across tumors studied to date. On the other hand blocking autophagy, or the cellular recycling of organelles and macromolecules via lysosomal degradation - which provides an alternative but finite source of arginine - may be more tractable using existing quinoline-based anti-malarials. Finally, the stromal compartment of tumors is also a potential source of arginine flux that may modulate the efficacy of arginine deprivation in the clinic. Thus, as our understanding of the arginine metabolome in tumorigenesis increases, it is hoped that further refinements of arginine degrading therapies, will translate to improved outcomes for patients with currently intractable cancers.
